# Diaphragmatic Neurophysiology and Respiratory Markers in ALS

**DOI:** 10.3389/fneur.2019.00143

**Published:** 2019-02-21

**Authors:** Mamede de Carvalho, Michael Swash, Susana Pinto

**Affiliations:** ^1^Instituto de Fisiologia-Instituto de Medicina Molecular, Faculdade de Medicina, Universidade de Lisboa, Lisbon, Portugal; ^2^Department of Neurosciences and Mental Health, Hospital de Santa Maria-CHLN, Lisbon, Portugal; ^3^Barts and the London School of Medicine, Queen Mary University of London, London, United Kingdom

**Keywords:** amyotrophic lateral sclerosis, diaphragm physiology, progression, respiratory function tests, survival

## Abstract

The main reason for short survival in amyotrophic lateral sclerosis (ALS) is involvement of respiratory muscles. Severe compromise of diaphragmatic function due to marked loss of motor units causes poor inspiratory strength leading to symptomatic respiratory fatigue, and hypercapnia and hypoxemia, often firstly detected while sleeping supine. Weakness of expiratory muscles leads to cough weakness and poor bronchial clearance, increasing the risk of respiratory infection. Respiratory tests should therefore encompass inspiratory and expiratory function, and include measurements of blood gases during sleep. Non-volitional tests, such as phrenic nerve stimulation, are particularly convenient for investigating respiratory function in patients unable to perform standard respiratory function tests due to poor cooperation or facial weakness. However, SNIP is a sensitive test when patients with bulbar involvement are able to perform the necessary maneuvers. It is likely that central respiratory regulation is disturbed in some ALS patients, but its evaluation is more complex and not regularly implemented. Practical tests should incorporate tolerability, sensitivity, easy application for regular monitoring, and prognostic value. Impending respiratory failure can cause increased circulating inflammatory markers, but molecular assessment of respiratory distress requires further study. In future, home-monitoring of patients with accessible devices should be developed.

## Introduction

Respiratory insufficiency (RI) in ALS usually emerges in the late stage of disease progression, although it may sometimes be the presenting feature (1, 2). Respiratory complications, especially hypoventilation (2), reduced bronchial clearance and lung infection (3) account for the majority of deaths in ALS. Mild respiratory involvement causes fatigue in daily-life activities and disruption of sleep, with negative impact on quality of life (4, 5) and hypoxemia may impair cognitive function (6), especially important in a population with a lower cognitive reserve.

ALS affects both inspiratory and expiratory muscles, as well as upper airway muscles (7). Cough, an essential reflex for airway protection and clearance, depends on effective glottis closure and efficient expiratory muscle function. Bulbar muscle dysfunction impairs the former and, for this reason, cough effectiveness is not always correlated with expiratory muscle weakness (8). Weakness of pharyngeal and laryngeal muscles increases the risk of aspiration and lung infection. The latter is more critical when associated with marked cough deficiency. For active inspiration the diaphragm is the most important muscle, although other muscles function as accessory muscles of inspiration, e.g., sternocleidomastoid, scalenus, trapezius, external intercostal, pectoralis, and paraspinal muscles. These are particularly important when the diaphragm is weak and during exercise. Severe diaphragm weakness leads to hypoxemia and carbon dioxide retention, since the work capacity of these accessory muscles is not sufficient to compensate. Furthermore, these muscles are themselves progressively involved in the disease process (2). In ALS, the major reason for frank respiratory failure is involvement of the diaphragm (2). The phrenic nerve motor nuclei in the cervical spinal cord are located in a region early affected in ALS, shown by early morphometric changes in these neurons (9). Dyspnea in ALS is closely correlated with diaphragmatic dysfunction (10). Indeed, diaphragm weakness as assessed by the evoked response to transcutaneous phrenic nerve stimulation is predictive of hypoventilation (11) and survival (12). It is therefore relevant to assess the physiology of the diaphragm in people with ALS.

## Diaphragm Physiology

The diaphragm is the most important muscle of ventilation. It is a dome-shaped muscle that separates the thoracic and abdominal cavities. It has a musculo-fibrous structure, formed by a central non-contractile fibrous region, and contractile muscle fibers that radiate circumferentially from the central tendon to attach peripherally to the upper three lumbar vertebrae posteriorly (crural diaphragm) and onto the inner surface of the lower six ribs and costal cartilages antero-laterally (costal diaphragm). In humans, the diaphragm comprises approximately equal numbers of type I and type II fibers, but these muscle fibers are smaller than in the expiratory muscles. They have a rich capillary supply and are resistant to aging (13). Muscle spindles are present only in small numbers in the diaphragm (14), so muscle stretching does not much modulate phrenic neuronal excitability. Diaphragm is well adapted to the rhythmic continuous periodical inspiration of ventilation and to ocassional more forceful contractions, as in deep breaths and coughing. The mean diaphragm thickness at the point of functional residual capacity is 2.29 ± 0.4 mm, as measured by ultrasound (15) but is variable over its surface, and also dependent on body position. Diaphragmatic thickness can increase two-fold during full inspiration (16).

The motor innervation of the diaphragm is almost exclusively from the phrenic nerve (C3–5), which branches to innervate the entire muscle. Contraction of the diaphragm causes axial descent of the dome of the muscle, decreasing intrapleural pressure, and increassing intrabdominal pressure, thus exerting an expansive force on the lower thorax (17). This negative intrathoracic pressure causes an inflow of air to the lungs, promoting inspiration. The diaphragm is a very mobile muscle. With full inspiration it flattens, expanding the thorax down to the level of costal margin anteriorly, and during forced expiration it rises anteriorly to the level of the fourth or fifth intercostal space.

There is appreciable force reserve in the diaphragm. In humans the maximum transdiaphragmatic pressure is about 11 kPa, which more than 10 times the value measured during eupnea (18). Indeed, normal respiration activates fatigue-resistant slow-units (19). However, coughing and sneezing are demanding maneuvers requiring very strong diaphragmatic contraction, close to 50% of the maximum transdiaphragmatic pressure, which implies activation of fast-fatigable motor units (19).

During calm breathing at rest expiration, unlike inspiration, is a passive phenomenon resulting from the relaxation of the inspiratory muscles and reduction of lung compliance. However, active forced expiration relies on recruitment of expiratory muscles, namely the internal intercostals and the abdominal ventro-lateral muscles (20). Generation of an adequate expiratory flux is needed for coughing, sneezing or vomiting. This is only possible with strong inspiration, closure of the glottis, and a sudden increase of intra-abdominal and intra-thoracic pressures. Effective peak cough flow (PCF) in healthy subjects exceeds 360–400 L/min (21). Peak flow values >160–200 L/min are needed for effective mucus expectoration (22) and values above 250–270 L/min are required to prevent aspiration pneumonia in patients with neuromuscular disorders (23).

The inspiratory pace-maker is located in the pre-Bötzinger Complex in the medulla (24). Its activity, both during inspiration and expiration, is modulated by inhibitory pre-motor neurons and by the Bötzinger Complex (18). Although expiration is a passive movement, active expiration involves a rostral generator, the retrotrapezoid nucleus (25). Synaptic drive to phrenic nerve nuclei is derived from pre-motor neurons located in the ipsilateral ventrolateral and dorsomedial medullary tracts, which respond to central chemoreceptors, sensitive to hypercapnia, and peripheral chemoreceptors, especially the carotid bodies, that are sensitive to hypoxemia. These premotor neurons are also sensitive to sleep-wake state modulation (18). Spinal interneurons can modulate phrenic motoneuronal activity, in particular via intercostal muscle afferents signaling strain of the chest wall (26). Voluntary control of breathing depends on fast, direct corticospinal inputs, which are also critical for respiratory control during speech (18). This pathway can be investigated by magnetic stimulation of cortical areas.

## Assessment of Respiratory Dysfunction in ALS

In ALS inspiratory and expiratory muscles, as well as upper airway muscles are progressively involved. Studies of a possible dysfunctional central respiratory drive are few, but it is likely this could be affected in some patients with ALS (27). As such, different tests are necessary to provide a global view of the respiratory function of diseased subjects. The American (28) and the European (29) guidelines agree that a first respiratory evaluation should be made at the baseline clinical assessment and then periodically thereafter. Nevertheless, this must be adjusted individually, according to the rate of progression of the disease and when there are intercurrent events, such as infection, that may affect respiratory function. A summary of the available tests, their utility and limitations is provided in Table 1.

**Table 1:** A summary of the most relevant respiratory tests in ALS.

	**Tolerability**	**Simplicity**	**Reliability**	**Sensitivity**	**Rate of change**	**Technical difficulty**	Cost^*^	Ease for monitoring^**^	**Experience in trials**
**GLOBAL**									
FVC	++	++	++	+	++	Volitional. Limited by orofacial paresis and dyscognition.	++	++	+++
SVC	++	+++	++	+	++	Volitional. Limited by orofacial paresis and dyscognition.	++	++	+++
MVV	+	+	+	+?	+?	Volitional. Needs motivation; Limited by orofacial paresis, fatigue, and dyscognition.	++	+?	0
NPO	+++	+++	+++	++	+	Limited by cold hands or poor sleep.	+	+++	0
TCP	+++	+++	+++	++	+?	Limited by cold hands and poor sleep.	++	+++	0
Sleep studies	+	0	+	+++	+	Limited by poor sleep.	+++	+	0
**INSPIRATORY TESTS**									
MIP	+	+	++	+++	+++	Volitional. Limited by orofacial paresis, fatigue, and dyscognition; early floor effect.	++	+	0
SNIP	++	++	++	++?	++	Volitional. Limited by orofacial paresis and dyscognition.	+	++	+++
Diaphragm US	+++	+++	+++	++?	++	Limited by dyscognition.	++	++	0
Phrenic stimulation	+	++	++	+	++	Limited by electrical stimulation intolerance.	++	++	
**EXPIRATORY TESTS**									
PEF	++	++	++	+?	++	Volitional. Limited by orofacial paresis and dyscognition.	++	++	0
PCF	++	+++	++	+?	++	Volitional. Limited by orofacial paresis and dyscognition.	+	++	0
MEP	+	+	++	+++	+++	Volitional. Limited by orofacial paresis, fatigue, and dyscognition; early floor effect.	++	+	0
**CENTRAL DRIVE FUNCTION**									
P01	++	+	+	+?	+?	Volitional. Limited by orofacial paresis, fatigue and dyscognition.	++	+	0

*FVC, forced vital capacity; SCV, slow vital capacity; MVV, maximal voluntary ventilation; NPO, nocturnal percutaneous oximetry; TCP, percutaneous capography; MIP, maximal inspiratory pressure; US, ultrasound; PEF, peak-expiratory flow; PCF, peak-cough flow; MEP, maximal expiratory pressure; P01, mouth occlusion pressure (100 ms)*.

^*^Cost (greater number of plus symbol means higher cost) was estimated taking into account equipament price and the requirement of a technician.

^**^Ease for monitoring was estimated considering patient confort and technical complexity.

### Global Respiratory Evaluation

Forced vital capacity (FVC) is a non-invasive respiratory test that has long been used in ALS. It assesses both the inspiratory and expiratory loops, requiring expiration done forcefully after a maximal inspiration, as opposed to slow vital capacity (SVC). This test is sensitive to change and predictive of hypoventilation and survival in ALS (30). The change of FVC is an adequate test to follow ALS patients (30, 31), since its decline rate tends to be linear (~3.5/month), there is a high interpatient variability (32) but this rate is a strong predictor of survival (33). FVC can be an unreliable measure of ventilatory function in patients with bulbar involvement due to orofacial weakness, due to air leakage around the mouthpiece (2). FVC is more sensitive in detecting diaphragmatic weakness when performed in the supine position (34), but this position is often poorly tolerated due to secretions or to the extent of diaphragmatic weakness. In addition, it is not a very sensitive test to detect hypercapnia, since gas exchange is well maintained until FVC values are very low (35). SVC is easier to perform in patients with bulbar involvement, because the air is exhaled slowly, with less air-leakage around the mouthpiece. SVC has been preferred in a number of recent trials, as it is very strongly correlated with FVC (and with other respiratory tests such as Maximal Inspiratory Pressure and Maximal Expiratory Pressure), as well as with ALSFRS-R (36). It is a predictor of progression, the need for positive pressure ventilation, and survival in ALS (37, 38).

Maximal voluntary ventilation (MVV) assesses respiratory function on maintained efforts. The patient is asked to breathe in and out, through a mounthpiece, as deeply and quickly as possible during 12 s, for at least two trials (39). The value is extrapolated for 1 min. The test is demanding for ALS patients, due to their respiratory fatigue. It can be a sensitive measure of disease progression (30), but only in the early stages of the disease (39). This test is rarely performed in daily practice.

Nocturnal pulse oximetry (NPO) is a useful, non-invasive, inexpensive, and convenient method, which accesses respiratory function in a demanding state—when patients are lying and sleeping. It can be used individually or during polysonography, the latter allowing for clear characterization of possible central and/or peripheral apnea. NPO assesses percutaneous oxygen saturation (maximum, median, and minimum values), in relation with heart rate. Further, the pattern of the oxygen saturation curve overnight can be explored. NPO has been shown to be predictive of survival in ALS (40, 41). In addition, it can indicate central drive dysfunction in patients with normal respiratory muscles, a factor that is probably more common in spastic patients (42). NPO is a mandatory method to follow non-invasive ventilation adaptation in patients, which permits home-telemonitoring and distance alteration of ventilatory settings (43). Transcutaneous capnometry (PtcCO2) is a more modern approach to evaluate respiratory function in ALS and other neuromuscular disorders (44). PtcCO2 recordings show strong correlation with arterial measurements. A value higher than 49 mmHg during ≥10% of the total recording time indicates respiratory insufficiency (44). Transcutaneous capnography has been strongly recommended for detection of nocturnal hypoventilation in patients with ALS (45). In patients on non-invasive ventilation, PtcCO2 can be helpful to monitor a proper ventilation, in particular to differenciate between hypoventilation and hypoxemia related to other reasons like as ventilation/perfusion mismatch, as well as in detecting hyperventilation (46). Both techniques have some limitations, for example they cannot discriminate other causes of sleep disturbances, such as obstructive sleep apnea, drug-effect, or associated lung disorder. Nonetheless they are very convenient as a screening method.

Blood gas measurements provide information about CO_2_ retention and hypoxemia when respiratory failure is severe. Because respiratory assessment is desiged to evaluate early changes, this test is not extensively used in ALS; however, it can provide relevant information for respiratory management in some patients.

Sleep studies have been investigated for a long time in ALS. In this disorder, reduction of the rapid eye movement (REM) sleep stage is typically observed, in particular when the diaphragm is markedly affected and accessory respiratory muscles are weak (47). It has been speculated that disturbed REM sleep might protect patients from hypoventilation (48). However, in patients with preserved diaphragmatic function, signs of sleep hypoventilation are observed as frequently in REM and non-REM phases (27), probably due to reduced respiratory drive (42). Arnulf et al. (48) found that ALS patients with upper motor neuron involvement to respiratory muscles tended to have abnormal REM sleep and poor prognosis. There is a strong link between severity of respiratory function impairment, poor quality of sleep, and daytime somnolence, in ALS (49).

### Evaluation of Inspiration

Maximal inspiratory pressure (MIP) and nasal inspiratory pressure during a maximal *sniff* (SNIP) are inexpensive and non-invasive respiratory measures that access maximal inspiratory muscular strength, the first against a mouth occlusion and the second using a plug inserted in one nostril (50–52). In both, it is necessary to secure cooperation from patients to breath forcefully against a resistance. While 3 consistent measures are necessary to determine MIP (53), the number rises to 10 for SNIP, 5 in each nostril (53, 54), as the result improves with practice. Fatigue is a limiting factor for both techniques. MIP is more sensitive than FVC in detecting hypoventilation (55). However, its marked early decline (floor effect) limits its use in following patients and it is difficult to perform in patients with orofacial weakness (56) or with spasticity. SNIP is a sensitive tool especially suited for ALS patients with orofacial weakness. It is predictive of survival (57) and of the onset of significant hypoventilation in spinal-onset patients (5). There is some uncertainty about the best technical approach to test ALS patients in order to obtain reliable values (58). SNIP seems to depend more on diaphragm force and MIP more on the sternocleidomastoid muscle power, making these tests complementary (59).

Transdiaphragmatic pressure (Pdi) can be assessed by inserting balloon catheters in the stomach and mid-esophagus and measuring the differential pressure during active maximal inspiration (60) or following stimulation of the phrenic nerve (61). This is an uncomfortable test that is not suited to clinical application.

Diaphragmatic ultrasound (US) is a non-invasive technique that assesses diaphramatic dynamics, and measures the muscle thickness at tidal volume and on maximal inspiration, as well as the ratio between baseline and maximal inspiration, useful measures to detect diaphragm involvement (62, 63). Significant correlations have been found between these measurements and FVC, SNIP, and the amplitude of the motor response of the phrenic nerve (62–64). However, ultrasound studies are less sensitive than phrenic nerve motor responses in assessing early deterioration of the diaphragm in ALS (65).

**Figure 1:**
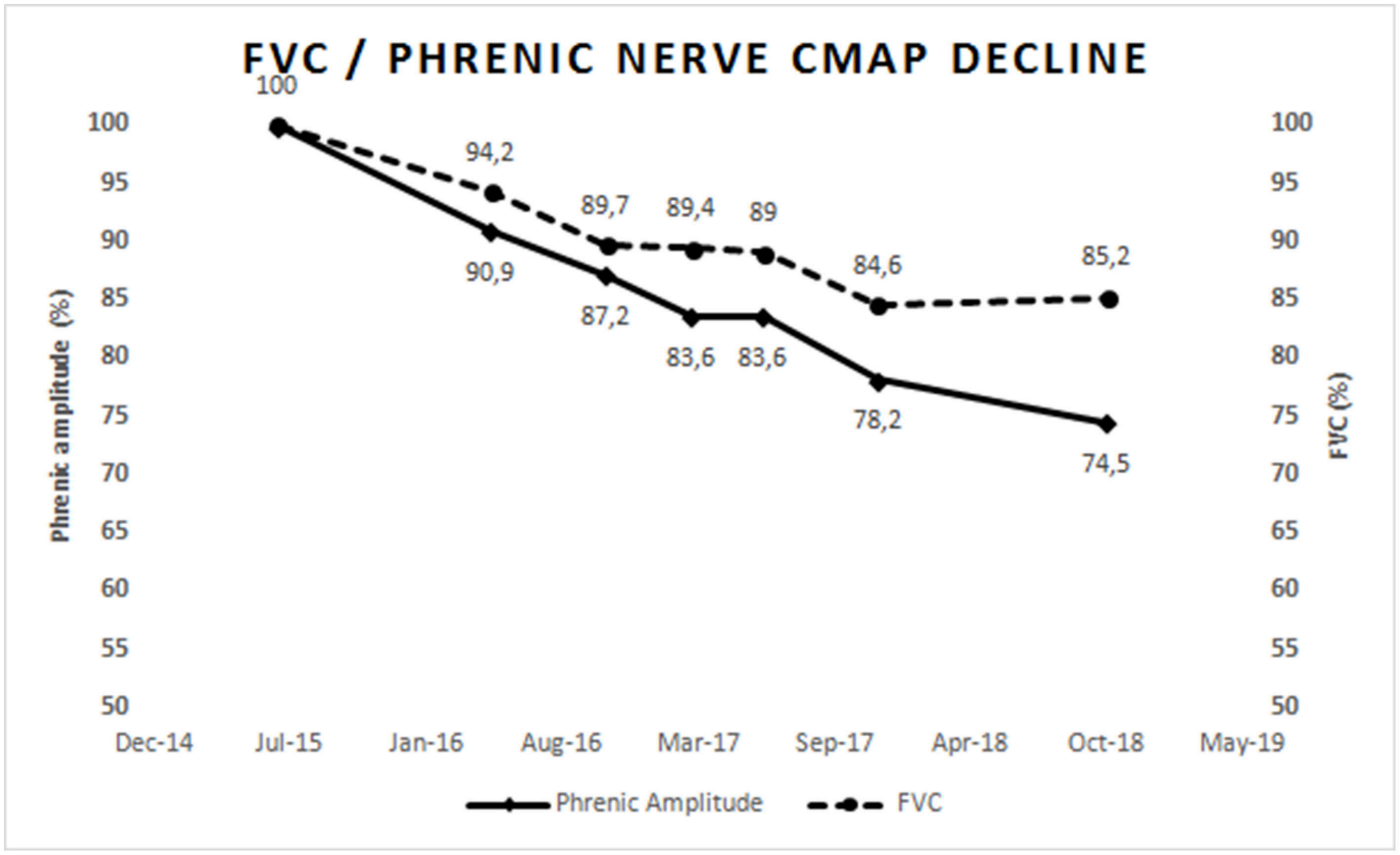
Represents the progressive parallel decline of FVC and phrenic nerve compound muscle action potential amplitude in an ALS patient with slow progression. Values were normalized to 100% of normal at first assessment. This figure is simply for representative purposes and not intended to present research findings.

Phrenic nerve stimulation by percutaneous electrical or magnetic stimulation in the neck to elicit diaphragm motor responses is an objective, non-volitional test (66, 67) that can be used to assess the number of functional motor units in the diaphragm (68). Abnormal amplitude (or area) of the motor response has good predictive value for hypoventilation in both bulbar- and spinal-onset patients, and is correlated to FVC (11). This technique is useful in patients with marked facial weakness or in those unable to cooperate, for example those with fronto-temporal dementia. The amplitude of the motor response declines significantly over 3–6 months, and correlates with FVC and SNIP change (69); it is predictive of survival in ALS (12). Figure 1 represents the progressive and parallel decline of FVC and phrenic nerve compound muscle action potential in an ALS patient.

### Evaluation of Expiration

The efficiency of the expiratory muscles can be easily addressed by evaluating the peak expiratory flow (PEF) and the peak cough flow (PCF), and maximal expiratory pressure (MEP) evaluates the strength of these muscles. These three volitional tests are simple to perform, inexpensive, and non-invasive. Although they measure expiratory muscle function, they depend on central motor control as well as on the efficiency of the inspiratory muscles. Abnormally reduced values indicate inability to expel bronchial secretions, leading to a high risk of respiratory infections (39), leading to increased morbility and mortality (70). MEP values are measured by asking the patient to exhale forcefully against an ocluded mouthpiece. Abnormal values are common in ALS patients (8, 71), and correlate with inspiratory involvement. PEF and PCF use peak flow meters, coupled with a face mask for PCF testing, and assess the ability to exhale forcefully after a maximal inspiration (72, 73) and to cough after a submaximal inspiration (60). Coughing can also be assessed by the gastric pressure generated during a maximal cough, which is a sensitive method to assess expiratory muscle strength, but this is an invasive and poorly tolerated test (74).

### Evaluation of the Central Respiratory Drive

Both NPO and sleep studies can detect respiratory center dysfunction, in particular in patients with normal respiratory muscles in whom nocturnal hypoventilation is detected without obstructive apnea (27). Inspiratory mouth occlusion pressure at 100 ms during quiet breathing (P0.1) is considered an indicator of respiratory drive. Spastic patients with normal diaphragm function tend to show abnormal P0.1/FVC values, associated with a poor prognosis for survival (42). P01 values are similar in bulbar and spinal-onset patients at presentation (56), suggesting that impaired central drive does not depend on the region of onset. The observation of “respiratory apraxia” in ALS patients highlights the complexity and importance of the cortical control of respiration and its potential involvement in ALS (75).

## Conclusions

There are many tests available to evaluate different features of respiratory function in ALS. In general, most centers follow a conventional approach by evaluating SVC and FVC, which are are often applied in clinical trials. Patients may also be asked to undergo maximal pressure measurements, expiratory peak flows and nocturnal oximetry, sometimes associated with EEG recordings. Less commonly, diaphgram ultrasound or phenic nerve motor responses to percutaneous cervical electrical stimulation of the nerve are tested. Percutaneous capnography is emerging as a relevant technique. Disparity in patients'tolerability and technical limitations would recommend to apply more than one single test to assess respiratory function in ALS patients.

A future study combining most of these tests in a single set of ALS patients would provide more information about diagnostic accuracy, sensitivity, realibility, and convenience for monitoring disease progression. This would have major potential implications in clinical trials, since changing the rate of respiratory decline is critical for improving survival and functional capability.

The identification of a molecular marker of respiratory impairment in ALS would be a convenient and valuable test. Some research indicates that respiratory insufficiency can precipitate an inflammatory response (76, 77), and this is a new avenue yet to be fully explored. User-friendly devices for in-home respiratory evaluation is another future step. New tests to directly evaluate strength of respiratory muscles will require a better understanding of their physiology.

## Author Contributions

All authors listed have made a substantial, direct and intellectual contribution to the work, and approved it for publication.

### Conflict of Interest Statement

The authors declare that the research was conducted in the absence of any commercial or financial relationships that could be construed as a potential conflict of interest.
